# Review of Bioactivity, Isolation, and Identification of Active Compounds from *Antrodia cinnamomea*

**DOI:** 10.3390/bioengineering9100494

**Published:** 2022-09-22

**Authors:** Hua-Xiang Li, Juan-Juan Wang, Chun-Lei Lu, Ya-Jun Gao, Lu Gao, Zhen-Quan Yang

**Affiliations:** 1 College of Food Science and Engineering, Yangzhou University, Yangzhou 225009, China; 2 Jiangsu Key Laboratory of Dairy Biotechnology and Safety Control, Yangzhou University, Yangzhou 225009, China

**Keywords:** *Antrodia cinnamomea*, bioactive, compound, isolation, purification

## Abstract

*Antrodia cinnamomea* is a precious and popular edible and medicinal mushroom. It has attracted increasing attention due to its various and excellent bioactivities, such as hepatoprotection, hypoglycemic, antioxidant, antitumor, anticancer, anti-inflammatory, immunomodulation, and gut microbiota regulation properties. To elucidate its bioactivities and develop novel functional foods or medicines, numerous studies have focused on the isolation and identification of the bioactive compounds of *A. cinnamomea*. In this review, the recent advances in bioactivity, isolation, purification, and identification methods of active compounds from *A. cinnamomea* were summarized. The present work is beneficial to the further isolation and discovery of new active compounds from *A. cinnamomea*.

## 1. Introduction

*Antrodia cinnamomea* (syn. *Antrodia camphorate*) is a precious edible and medicinal mushroom; it belongs to phylum Basidiomycetes, family Polyporaceae, and genus *Antrodia* [1,2]. This fungus grows only on the inner cavity of a native tree called *Cinnamomum kanehirai* Hayata at an extremely slow pace and has been known as “ruby in the forest” [3,4]. *A. cinnamomea* has been historically used to treat food and drug intoxication [5]. *A. cinnamomea* exhibits various physiological and pharmacological properties, such as anticancer, antitumor, antioxidant, anti-inflammatory, hypoglycemic, hepatoprotective, immunomodulation, and gut microbiota regulation activities [6,7,8,9]. It has also attracted increasing research attention for the development of novel medicine due to its therapeutic action, particularly its prospective application as a chemoprophylaxis agent [1].

However, the wild fruiting bodies of *A. cinnamomea* are extremely expensive and in short supply because of host scarcity and slow growth. Accordingly, artificial cultivation techniques, such as cutting wood culture, solid-state fermentation, submerged fermentation, and dish culture, have been developed to supplement the expanding demand for *A. cinnamomea*. However, obtaining adequate amounts of excellent-quality *A. cinnamomea* through artificial culture has been greatly challenging [10,11]. Several differences exist between the type and content of the fruiting bodies and cultured mycelia of *A. cinnamomea*. Accordingly, numerous studies have demonstrated various methods for bridging such a disparity in bioactive metabolites between fruiting bodies and cultured mycelia [10,12].

With further in-depth studies, the research on *A. cinnamomea* is no longer limited to crude extracts. Various single substances, including polysaccharides, triterpenoids, ubiquinone derivatives, and maleic and succinic acid derivatives, have been isolated from fruiting bodies and cultured mycelia of *A. cinnamomea*. Currently, numerous kinds of isolation methods are used for the metabolites from *A. cinnamomea*. However, the standardization of product quality and purity is lacking. Numerous active substances remain undiscovered due to the limitations of isolation and purification methods. In this review, we summarized the recent findings on the active compounds of *A. cinnamomea*, their physiological and pharmacological properties, and methods of isolation and purification of active compounds.

## 2. Isolation and Purification of Bioactive Compounds from *A. cinnamomea*

**Figure 1:**
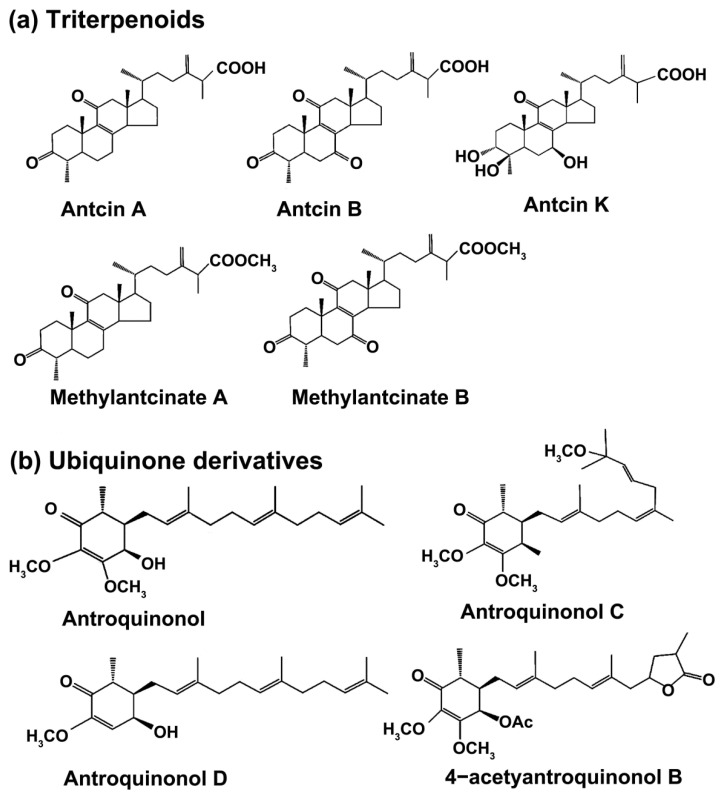

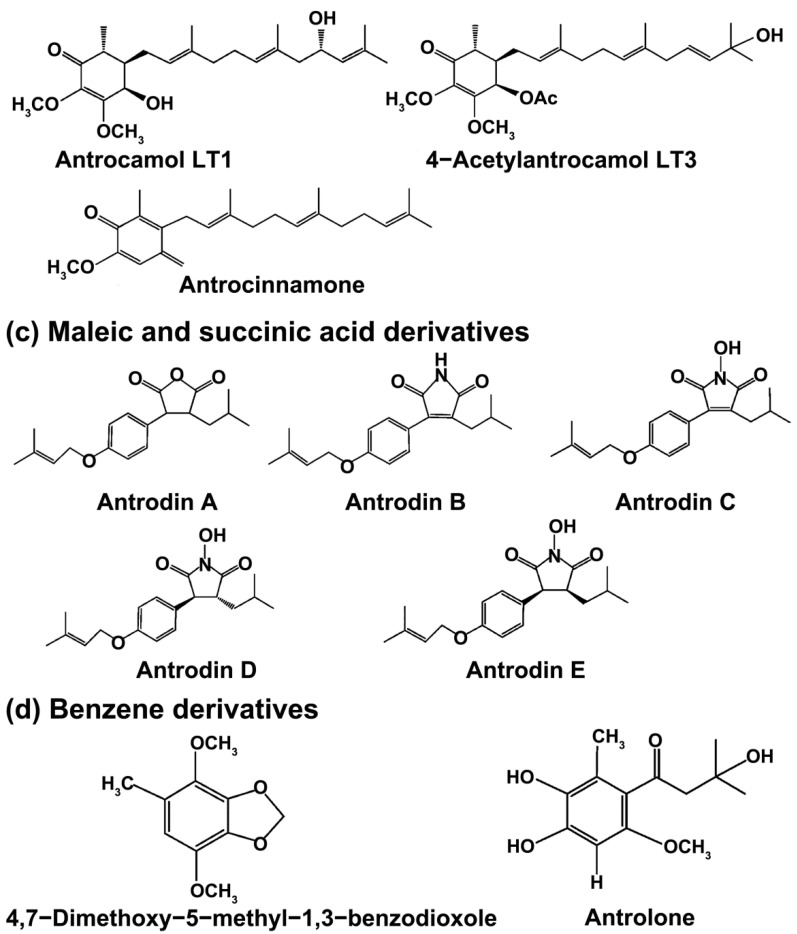
The chemical structures of the main bioactive components from *Antrodia cinnamomea*. (**a**) Triterpenoids, (**b**) ubiquinone derivatives, (**c**) maleic and succinic acid derivatives, (**d**) benzene derivatives.

More than 200 compounds have been isolated and identified in *A. cinnamomea*, and they include polysaccharides, triterpenoids, ubiquinone derivatives, maleic and succinic acid derivatives, benzene derivatives, and glycoprotein. The chemical structures of the main bioactive components from *A. cinnamomea* are showed in Figure 1. However, a number of compounds with significant biological activity in *A. cinnamomea* still have not been found [13,14]. The following section summarizes the separation and purification methods of various active substances from *A. cinnamomea*.

### 2.1. Polysaccharides

Polysaccharides are the predominant active constituents of *A. cinnamomea*, and they have great development potential and research value. These polysaccharides reportedly possess immunomodulatory, anti-inflammatory, anti-angiogenic, and anticancer activities [15,16]. Furthermore, polysaccharides are potential natural medications for the treatment of Parkinson’s disease [17,18].

The bioactive polysaccharides from *A. cinnamomea* are primarily obtained by submerged fermentation, and numerous studies have been performed to isolate additional varieties of polysaccharides due to their biological and application value. Polysaccharide separation is a complex procedure. The first step involves obtaining crude polysaccharides using water extraction and ethanol precipitation with an ethanol concentration of 70–80%. After crude extraction, several impurities, especially proteins, remain. The common methods of protein removal include the Sevag [19], trichloroacetic acid [20], and enzymatic methods [21]. The enzymatic method is also suitable for the removal of nucleic acid impurities. The treatment of protease K, DNase I, and RNase can conveniently and efficiently remove proteins and nucleic acids [21]. Crude polysaccharides often contain dark impurities that require decolorization, typically with 10% H_2_O_2_ [22], through the use of oxidants to destroy the chromogenic groups of pigments. Other impurities, such as monosaccharides, oligosaccharides, inorganic salts, and low-molecular-weight nonpolar substances, can be removed using dialysis. After impurity removal and decolorization, partially purified polysaccharides need further separation to yield the major pure polysaccharides.

Column chromatography is currently the most common technology for polysaccharide purification due to its high purification efficiency and simple operation. Ion-exchange column chromatography and gel column chromatography are the best known methods for the purification of polysaccharides from *A. cinnamomea*. For bulky polysaccharide solutions, ion-exchange column chromatography is often utilized initially. This method may be used to concentrate and preliminarily purify polysaccharide solutions, and certain polysaccharides can be purified as homogenous fractions. Diethylaminoethyl (DEAE)-cellulose, DEAE-Sephadex, and DEAE-Sepharose are the most often used anion exchangers [23], with DEAE-cellulose being the primary choice for the purification of polysaccharides from *A. cinnamomea*. Gel column chromatography is used to separate polysaccharides based on the size and shape of polysaccharide molecules. Anion-exchange column chromatography is generally used to undertake preliminary purification of the resulting crude polysaccharides, followed by gel column chromatography for further purification. Sephadex G-100 is a commonly used gel to purify polysaccharides from *A. cinnamomea*. Size-exclusion chromatography (SEC) is suitable for separating polysaccharides using aqueous solution as mobile phase and for the separation of water-soluble samples. Gel filtration chromatography (GFC) is also suitable for the separation of polysaccharides, and it uses different concentrations of salt and buffer solutions as eluents to separate and purify polysaccharides. However, GFC is unsuitable for mucopolysaccharide separation.

*A. cinnamomea* contains a mixture of polysaccharides composed of various monosaccharides [18]. Researchers have isolated numerous polysaccharides with different monosaccharide compositions and combinations that affect active functions. Perera et al. [21] reported presence of galactomannan (ACP) in *A. cinnamomea* mycelia for the first time. They observed that the compound is composed of 75% mannose and 25% galactose. ACP improves the phagocytosis and bactericidal activity of J774A.1 murine macrophages against *Escherichia coli* [21]. Further investigation revealed that ACP has immunostimulatory action via protein kinase C-α and mitogen-activated protein kinase phosphorylation in mouse peritoneal macrophages and human dendritic cells [24]. ACP2 is a galactoglucan derived from *A. cinnamomea* mycelia and mostly comprises glucose, galactose, and 6-deoxyglucose at a molar ratio of 5:2:1, and it reduces lipopolysaccharide-induced hepatocyte inflammation. The uniqueness of ACP2 is attributed to the 6-deoxyglucose in its sugar chain, which has seldom been documented in earlier works [25]. A purified polysaccharide that accounts for 40.96% of crude polysaccharides termed ACPS-1 is composed primarily of mannose, xylose, arabinose, fucose, and rhamnose at a molar ratio of 31.27:1.77:1.44:1.34:1.00, showing a strong in vitro inhibitory activity against various mouse and human cancer cell lines [19].

When polysaccharides are sulfated, their bioactivities reportedly increase. Compared with polysaccharides, sulfated polysaccharides have stronger biological activity for inhibiting cancer cells, inflammation, and angiogenesis [26,27,28]. Several studies revealed that the sodium sulfate-, ammonium sulfate-, and zinc sulfate-feeding of *A. cinnamomea* can increase the content of sulfated polysaccharides [29,30]. Sulfated polysaccharides are extracted differently from polysaccharides by utilizing the Albano and Mourao method with several modifications [31]. The lyophilized mycelia (1 g) is extracted with 40 mL of 0.1 M sodium acetate (pH 5.5) containing 5 mM of cysteine, 100 mg of papain, and 5 mM of ethylenediaminetetraacetic acid (EDTA) at 60 °C for 24 h. After obtaining crude sulfated polysaccharides, a purification method similar to that for polysaccharides is performed. The preparation procedures of polysaccharides from *A. cinnamomea* in recent publications are listed in Table 1.

### 2.2. Triterpenoids

The triterpenoids isolated from *A. cinnamomea* primarily include the ergostane and landostane types [34]. About 75 ergostane and 28 landostane have been reported in previous research [35]. Lanostanes are produced in fruiting bodies and cultured mycelia, whereas ergostanes are produced only in fruiting bodies [36]. The majority of triterpenoids have significant therapeutic potential. The latest research showed that triterpenoid-enriched extracts from cultured mycelia also attenuate alcohol-induced chronic liver injury [37]. Antcins include a group of ergostane-type triterpenoids in *A. cinnamomea*.

These natural substances are novel peroxisome proliferator-activated receptor α activators and may be potential chemicals for the improvement of current antidyslipidemic medicines [38]. Nearly 20 antcins have been separated to date [39]. Antcins A, B, C, H, and K are abundant in *A. cinnamomea*. Only antcin A has anti-inflammatory properties, which it exerts through imitating glucocorticoids. The extra carbonyl or hydroxyl group added to C-7 of the molecule distinguishes antcin A from the other four antcins [39]. The most abundant landostane-type triterpenoids are eburicoic acid, sulfurenic acid, and dehydrosulphurenic acid. All of them show a significant antidiabetic activity [40,41,42].

Triterpenoids have considerable physiological activity. Thus, the research on their separation and purification techniques is a great concern. Organic solvents are extensively applied to extract crude triterpenoids from *A. cinnamomea*, with the principle based on the polarity of each component differing between the fruiting bodies and cultured mycelia. The organic solvent-extraction method is beneficial to removing high-polarity substances and retaining low-polarity triterpenoids. In the selection of organic solvents, numerous researchers have used methanol and ethanol to obtain crude triterpenoid extract. *A. cinnamomea* includes a complex array of tetracyclic triterpenoids with extremely similar structures but varied number, position, or stereochemistry of hydroxyl groups, thus resulting in their difficult separation [43]. Most ergostanes are highly soluble in aqueous methanol, but lanostanes can be dissolved only in pure methanol due to their low polarity. Consequently, several researchers extracted ergostanes using 50% methanol and subsequently extracted the lanostane-enriched extract with 100% methanol. This straightforward method effectively separates the two forms of triterpenoids [43]. Triterpenoids are further isolated and purified by a traditional method called multistage solvent extraction. Its principle is similar to that of organic solvent extraction, except that multistage solvent extraction is repeatedly extracted using several organic reagents with different partition coefficients to extract purer substances as much as possible. However, obtaining pure compounds by multistage solvent extraction is difficult and causes solvent wastage, environmental pollution, and solvent residues in products. At present, multistage solvent extraction is generally used as an auxiliary method and combined with macroporous resin column chromatography, silica-gel column chromatography, and other techniques. Given its ease of use, low cost, and repeatability, macroporous resin column chromatography is extensively used to extract and purify natural materials. It may also be used to enhance a wide range of compounds [44]. AB-8 resins are preferred for triterpenoid purification because of the similar polarity of triterpenoids to that of AB-8 macroporous resin with a weak polarity, which enables its easy elution. Silica-gel column chromatography is suitable for the purification of triterpenoids. Different eluents are used for elution in the process to achieve separation and fractionation. Common solvent systems are the dichloromethane-methanol solvent system and chloroform-methanol solvent system, and triterpenoids can be separated by repeated silica gel column chromatography [45,46]. The preparation procedures of riterpenoids from *A. cinnamomea* in recent publications are summarized in Table 2. Ergostanes are tetracyclic triterpenoids that often exist as 25R/S epimeric pairs, making chromatographic separation challenging. Seven pairs of 25R/S-ergostanes were separated from *A. cinnamomea* using analytical supercritical-fluid chromatography (SFC) [47]. SFC is an effective approach for chiral and achiral separation. Its quick column equilibration and easy mobile phase removal make it appealing for preparative scale separation. SFC is commonly used to separate chiral combinations of pharmaceutical and natural compounds, such as triterpenoids, steroids, and bile acids [47]. In the separation and purification of triterpenoids, not only a single method is usually used but also multiple methods that supplement each other. A total of 60 triterpenoids, including 18 novel ones (antcamphins M–X), were isolated from dish cultured *A. cinnamomea* in accordance with the following methods. Dried dish-cultured samples of *A. cinnamomea* were extracted with 95% ethanol. Then, the extract was repeatedly subjected to AB-8 macroporous resin, silica gel, ODS C18, and Sephadex LH-20 column chromatography and further purified by semipreparative reversed-phase high-performance liquid chromatography (HPLC) [45]. The yield was the highest amount of triterpenoids isolated from *A. cinnamomea* at one point. However, the production of each triterpenoid is low. Triterpenoids are potential therapeutic agents in the medical industry; thus, the triterpenoid yield and extraction efficiency need improvement [38].

### 2.3. Ubiquinone Derivatives

Ubiquinone derivatives are important bioactive chemicals in *A. cinnamomea*, and they have significant anticancer and anti-inflammatory potential. To date, 13 kinds of ubiquinone derivatives, including antroquinonol, antroquinonol B, antroquinonol C, antroquinonol D, antroquinonol L, antroquinonol M, antroquinonol Z, antrocamol LT1, antrocamol LT2, antrocamol LT3, 4-acetyantroquinonol B, 4-acetylantrocamol LT3, and antrocinnamone, have been isolated from *A. cinnamomea* [51,52].

Antroquinonol is the first identified ubiquinone compound from the solid-state fermentation cultured mycelia of *A. cinnamomea* [53]. It was extracted with n-hexane and showed various bioactivities, such as anticancer, immunosuppressive, and diet-induced-obesity amelioration [53,54,55]. Silica-gel column chromatography (eluted sequentially with mixtures of n-hexane and ethyl acetate in a stepwise gradient mode) and gel column chromatography (an open column of Sephadex LH-20 with 95% ethanol as eluent) are used to purify antroquinonol [56]. However, almost no antroquinonol is obtained under liquid-state fermentation conditions [57]. When ubiquinone 0 is used as a precursor in liquid-state fermentation, antroquinonol production can be successfully induced [58]. Given the low content of antroquinonol in *A. cinnamomea*, the preparative isolation of antroquinonol remains a difficult process. The typical separation method is based on the repeated elution by silica-gel column chromatography, which is time consuming and tiresome. Meanwhile, valuable samples are always permanently adsorbed onto the silica gel, resulting in their wastage. Furthermore, the excessive use of organic solvents is harmful to the environment. However, as a liquid–liquid partition chromatography, high-speed countercurrent chromatography (HSCCC) may use a liquid stationary phase without a solid support, thereby avoiding irreversible adsorption and benefiting complete sample recovery [59,60]. HSCCC has been successfully used to separate the antitumor compound antroquinonol from the solid-state cultured mycelia of *A*. *cinnamomea*, and the purity of the isolated antroquinonol was 97.12%. This method can avoid irreversible stationary-phase adsorption and tedious and time-consuming separation steps [61].

4-Acetylantroquinonol B is isolated because of its excellent antiproliferative activity against HepG2 cells. Following mycelial ethanol extraction, silica-gel chromatography, and HPLC purification, 4-acetylantroquinonol B with a molecular weight of 462.6 g/mol has been discovered [62,63]. Three novel ubiquinone compounds from *A. cinnamomea* mycelia, including antrocamol LT1, antrocamol LT2, and antrocamol LT3, have been identified. After 95% ethanol extraction and CH_2_Cl_2_ separation, these ubiquinone derivatives have been obtained by multiple silica-gel chromatography under different conditions. They all exhibited selective cytotoxicities against five human cancer cell lines (CT26, A549, HepG2, PC3, and DU-145) with half maximal inhibitory concentration values ranging within 0.01–1.79 µΜ [64]. A further study revealed the presence of antrocinnamone and 4-acetylantrocamol LT3 in the cultured mycelia of *A. cinnamomea* for the first time. These new ubiquinone derivatives showed a relative toxicity against three human cancer cell lines. Compared with a previous study, the CH_2_Cl_2_-soluble fraction was subjected to column chromatography and HPLC separation [65]. In the following research, the 20 g of the CH_2_Cl_2_-soluble fraction was subjected to chromatography over silica gel and successively eluted with n-C_6_H_14_–CH_2_Cl_2_ (1:4), CH_2_Cl_2_, and CH_2_Cl_2_–MeOH (95:5) to generate five fractions. The third fraction was applied to silica gel column chromatography, eluted with CH_2_Cl_2_–acetone (95:5) to yield 324.0 mg of 4-acetylantrocamol LT3 [66]. The main preparation procedures of ubiquinone derivatives from *A. cinnamomea* are listed in Table 3.

The purification methods of ubiquinone derivatives from *A. cinnamomea* also primarily include preparative liquid chromatography and semi-preparative liquid chromatography [66,67]. They are fast and effective tools for the analysis and separation of ubiquinone derivatives from *A. cinnamomea*. Compared with other methods, such as SFC, the equipment of liquid chromatography and semi-preparative liquid chromatography are simpler and less costly, and no energy is required to cool or heat carbon dioxide [68].

### 2.4. Maleic and Succinic Acid Derivatives

The maleic and succinic acid derivatives found in *A. cinnamomea* are natural products with the best hepatoprotective activity after silymarin [69]. Antrodins A–E are the crucial maleic and succinic acid derivatives of *A. cinnamomea*, and they exert substantial cytotoxic effects on Lewis lung carcinoma (LLC) tumor cell lines [70]. Studies have revealed that maleic acid derivatives exert a protective effect on liver injury. Antrodin A can increase the antioxidant and anti-inflammatory capabilities of the liver, making it a promising candidate chemical for acute alcoholic liver damage [71]. Antrodin B inhibits transforming growth factor (TGF)-1-induced cell proliferation, migration, and extracellular matrix (ECM) buildup in vitro, resulting in anti-hepatofibrotic action [72]. Antrodin C suppresses hepatic stellate cell activation, migration, and ECM production LLC partially through the inhibition of the platelet-derived growth factor and TGF-β1 signaling pathways, which are the two most potent stimuli of liver fibrosis [73]. Antrodin C also inhibits breast cancer cell migration and invasion by suppressing the Smad2/3 and β-catenin signaling pathways [74]. Apart from antrodins, maleimide, and maleic anhydride derivatives (antrocinnamomins A–D) show good inhibitory effects on nitric oxide generation [75].

In 2004, five maleic and succinic acid derivatives were isolated for the first time from cultured mycelia of *A. cinnamomea* [70]. CHCl_3_ was used to extract the powdered mycelia for 3 h under reflux. For further separation, CHCl_3_ extract was chromatographed on a silica gel and eluted with n-hexane-acetone (19:1–14:6) and CHCl_3_–MeOH (1:1) to yield nine fractions. They were then chromatographed on normal and reversed-phase silica gel to obtain five new maleic and succinic acid derivatives. Two maleic and succinic acid derivatives were a mixed in one fraction and subsequently separated by preparative HPLC [column: Tosoh TSK-gel ODS-80T_M_ (21.5 × 300 mm^2^), mobile phase: CH_3_OH–H_2_O containing 0.1% trifluoroacetic acid (70:30)] [70].

Later studies have confirmed five maleic and succinic acid derivatives, which were named antrodins A–E [76]. Researchers have optimized their separation procedures due to their significant bioactivity. Given that chloroform is poisonous, antrodins are extracted with methanol or ethanol rather than chloroform. Dried and crushed cultured mycelia of *A. cinnamomea* is extracted with 95% ethanol and then condensed at decreased pressure. The residues are suspended in water before being partitioned with n-hexane and ethyl acetate. Antrodin C is isolated from the ethyl acetate fraction by subsequent chromatography on silica gel and Sephadex LH-20 columns [77]. The semipreparative HPLC may increase the purity of antrodin C. A semipreparative HPLC column (Waters XBridge C18 column, Φ19 × 250 mm^2^, 5 µm) is used to separate crude antrodin C. The mobile phase comprises distilled water H_2_O (0.5% acetic acid) and acetonitrile (34851, Sigma–Aldrich) (10 mL/min) to obtain antrodin C with a purity exceeding 95% [73]. The mixture of antrodins A and B is separated with a semipreparative HPLC column (XBridge C18 column, 19 mm × 150 mm, 5 µm; Waters, Milford, MA, USA). For the acquisition of antrodins A and B, the mobile phase is composed of H_2_O (0.5% acetic acid) and acetonitrile at a flow rate of 7.2 mL/min, and their content is only 0.004% and 0.001%, respectively [72]. The preparation procedures of maleic and succinic acid derivatives from *A. cinnamomea* in recent publications are summarized in Table 4.

### 2.5. Benzene Derivatives

Several benzenoid compounds from *A. cinnamomea* possess potent anti-inflammatory properties. 4,7-Dimethoxy-5-methyl-1,3-benzodioxole (DMB) is a representative benzenoid compound and was first discovered in the crude methanol extract of *A. cinnamomea* fruiting bodies and cultured mycelia. Previous research has revealed that DMB exerts a significant anti-inflammatory properties by inhibiting superoxide production in human neutrophils. Furthermore, DMB therapy reduces dendritic cell-mediated Th2 allergic responses [78]. Antrolone, another benzenoid compound from *A. cinnamomea* mycelia, has an excellent anti-inflammatory effect, indicating its possible use in the development of novel anti-inflammatory drugs [79]. Coenzyme Q_0_ (2,3-dimethoxy-5-methyl-1,4-benzoquinone) is another important benzenoid compound of *A. cinnamomea*, and it possesses antitumor and anti-inflammatory activities [80,81,82].

At present, a limited number of studies have been conducted on the isolation and purification of benzenoid compounds. Shie et al. [83] reported a method for the isolation of DMB from the cultured mycelia of *A. cinnamomea*. Briefly, freeze-dried and powdered mycelia are extracted with methanol and then filtered. The filtrate is concentrated by evaporation under decreased pressure to produce the methanol extract. The extract is suspended in distilled water, and the aqueous solution is partitioned with n-hexane, ethyl acetate, and n-butanol in sequence. The ethyl acetate layer is evaporated to dryness, and the residue is chromatographed on a silica gel with n-hexane:ethyl acetate (7:1) to elute a crude fraction containing DMB. For the acquisition of pure DMB, this fraction is purified using a Sephadex LH-20 column with methanol as the eluent [83]. The DMB concentration is so low that just 4.79 g can be extracted from 5 kg of freeze-dried mycelia [83]. In addition, in accordance with Yen’s research [79], antrolone is extracted with 95% ethanol to yield the crude extract. The crude extract is dissolved in water and partitioned thrice with chloroform after condensation under a low pressure. To further purify antrolone, scientists subject the chloroform fraction to silica-gel column chromatography and medium-pressure liquid chromatography [79]. The preparation procedures of DBM and antrolone from *A. cinnamomea* in recent publications are summarized in Table 5.

### 2.6. Gycoproteins

According to reports, minimal variations in dextran glycosidic linkages, high-order structure, molecular weight, solubility, protein and lipid side chains, and/or high-order aggregates can result in significant differences in bioactivity [84,85]. Chiu et al. [86] first isolated a unique protein-bound polysaccharide with a molecular weight of 442 kDa from the submerged fermentation mycelium of *A. cinnamomea*. This polysaccharide is identified as an antrodan with a complex union of α- and β-glucans, which have (1 → 4)-linked α-Glcp and (1 → 3)-linked β-Glcp linkages to the carbohydrate chains though the asparagine connection with protein sites. Antrodan at low dosages (40 mg/kg) may be a potential hepatoprotective agent. However, increased doses of antrodan may cause certain unpleasant responses [87]. Antrodan is also useful in lung-cancer treatment and exerts antimetastatic effects [88,89]. Furthermore, when combined with cisplatin, antrodan substantially alleviates cisplatin-induced renal impairment. Antrodan has also been successfully used to treat non-alcoholic fatty liver disease through the AMP-activated protein kinase/Sirt1/peroxisome proliferator-activated receptor-γ/sterol receptor element-binding protein 1c pathway [90].

Glycoprotein is a binding protein and a complex polysaccharide. Thus, it has certain properties of proteins and polysaccharides. Most glycoproteins are soluble in water, dilute salt, and dilute alkali solution. Therefore, the extraction method of proteins or polysaccharides can be used based on its properties. A glycoprotein named antrodan was isolated from cultured mycelia of *A. cinnamomea* [86]. The researchers initially isolated polysaccharide AC-2 by alkali extraction with subsequent acid precipitation. Then, the free sugars and amino acids were dialyzed for three days with deionized water (DDW). The antrodan extracts were placed onto a Sepharose CL-6B column (3.0 × 82 cm^2^) and eluted with DDW at pH 11.0 (adjusted using 1 M NaOH) to separate polysaccharides at a flow rate of 0.5 mL/min. The target was collected with a fraction collector. High-performance SEC yielded the product at roughly 10% with an average molecular weight of 442 kD. The protein content of antrodan was 71.0%, which was evidently larger than the carbohydrate quantity (14.1%); thus, this antrodan was classified as a glycoprotein [86]. The main preparation procedures of glycoprotein from *A. cinnamomea* are listed in Table 6.

## 3. Identification and Quantification of Bioactive Compounds from *A. cinnamomea*

Aside from isolation and purification, the identification of particular bioactive compounds of *A. cinnamomea* is critical. This knowledge is essential for understanding the biological activities of these metabolites and discovering new *A. cinnamomea* bioactive compounds. Typically, the characterization and quantification of natural products can use various detection techniques, including nuclear magnetic resonance (NMR), infrared spectra, and mass spectrometry (MS) detection. NMR has steadily developed and has become an essential technology for chemical identification and structural characterization. This technique enables the acquisition of data from extremely mass-limited samples and the investigation of very complex materials [91]. ^1^H-NMR and ^13^C-NMR analyses were used to identify compounds from solid-state cultured mycelia of *A*. *cinnamomea* [92]. Finally, a quinone, four phenolic acid derivatives, three ubiquinone derivatives, two alkaloids, and a triterpenoid has been identified. These compounds exhibit potent neuroprotective activities against 6-hydroxydopamine-induced toxicity in PC12 cells [92]. NMR can also be used to determine the amounts of various components in a mixture quickly and precisely. This method, known as quantitative NMR (qNMR), has found novel uses in biological and pharmacological research. qNMR is a primary ratio method compared with other instrumental analytical methods because the resulting peak areas are proportionate to the number of matching nuclei. Consequently, qNMR has been selected for the quantitative analysis of a benzenoid-rich fraction containing three primary benzenoids of *A. cinnamomea* due to its superiority to other standard chromatographic techniques in detecting the amounts of specific herbal mixture elements [93].

Fingerprint analysis is used for the thorough assessment of the quality of herbal medicines and their associated products. Recently, the HPLC fingerprinting approach supplemented by ultraviolet (UV) and photodiode array (PDA) has been widely utilized for herbal-quality testing. However, UV and PDA detectors present several problems in the isolation of target analytes from interfering impurities. Thus, these techniques involve time-consuming sample preparation and long HPLC run times. To resolve these issues, scientists use the more sensitive liquid chromatography–tandem MS (HPLC-MS/MS), which can offer better overall information, such as molecular weight, retention time, and analyte collision fragments. Accordingly, a validated HPLC-MS/MS method for the quick and precise measurement of compounds from *A. cinnamomea* was established [94]. The method can be used to simultaneously measure seven characteristic chemicals, including antcin A, antcin B, antcin C, antcin H, antcin K, dehydroeburicoic acid, and DMB [94]. Triterpenoids are a significant bioactive component of *A. cinnamomea*, and different cultivation techniques exhibit significant variations. Qiao et al. [43] reported the contents of 10 ergostane-type and 8 landostane-type triterpenoids of *A. cinnamomea* samples derived from cutting wood culture, solid-state fermentation, submerged fermentation, and dish culture. It was determined by ultra-high performance liquid chromatography/ultraviolet (UPLC/UV) or supercritical fluid chromatography coupled with mass spectrometry (SFC/MS, for 25R/S-antcin A) within 16 min. The result showed that the 18 kinds of triterpenoids accounted for 118.2 ± 28.2, 89.4 ± 30.8, and 116.5 ± 1.1 mg·g^−1^ in wood-cultured fruiting bodies, wood-cultured mycelia, and dish-cultured, respectively. However, no triterpenoids were detected in the solid support cultivation or submerged fermentation samples in this research [43]. The development of products derived from *A. cinnamomea* benefits from the precise and rapid measurement of signature compounds in various samples using this method. Ultra-performance liquid chromatography quadrupole time-of-flight MS (UPLC/Q-TOF/MS) has been used to characterize and quantify mixture compounds. For the first time, the UPLC/Q-TOF/MS method was used to identify the extracts from *A. cinnamomea* and led to the discovery of 139 chemical compounds, including 102 terpenoids, 8 benzenoids, 2 purine nucleosides, and 27 other classes [95]. The development of this method has significant implications for the exploration of *A. cinnamomea* products.

## 4. Summary and Future Perspectives

Pharmacologically active natural compounds have achieved extraordinary appeal in recent decades, and certain bioactive molecules have historically made significant contributions to medication development. *A. cinnamomea* as a burgeoning medicinal fungus has multiple significant biological activity and pharmacological functions. Researchers have isolated and purified various bioactive compounds from its fruiting bodies and cultured mycelia. Despite recent advances, critical concerns regarding the fundamental research and use of *A. cinnamomea* remain unresolved.

First, obtaining active substances from *A. cinnamomea* by conventional solvent extraction has a number of drawbacks, particularly solvent wastage and low extraction efficiency. The extensive use of organic solvents is bound to cause pollution and harm people’s health. Thus, the identification of novel solvents to replace traditional organic solvents is necessary. Alternative solvents aim to provide an extraction method that is environment friendly and of high quality [96]. Researchers still lack a process for the systematic identification of characteristic metabolites of fruiting bodies and artificially cultured mycelia of *A. cinnamomea*. The current patterns in isolation, purification, and identification of characteristic metabolites are the bottlenecks in the development of high-quality *A. cinnamomea* products. Optimizing such isolation and purification methods is necessary to improve the product quality.

Second, the low content of *A. cinnamomea* active constituents inhibits their development in the field of medicine. Apart from improving the efficiency of isolation and purification, the yield of bioactive compounds should be increased. Moreover, waste is inevitable in the extraction of active substances. China produces about hundreds of metric tons of *A. cinnamomea* extraction waste every year [97]. Interestingly, this waste as feed supplement in the aquaculture industry increases the feed efficiency of zebrafish remarkably and reduces symptoms of inflammatory diseases in fish [97]. The reuse of extraction waste realizes the recycling of *A. cinnamomea* resources and reduces environmental pollution, thereby providing an accurate research idea to further study the use of extraction residue and improve its utilization value and paving the way for green sustainable development in the future.

Finally, the mechanisms underlying the effects of certain active compounds from *A. cinnamomea* remain unclear. One of the main reasons for this dilemma is that researchers usually use extracts or mixtures rather than pure compounds to test the effect, which causes difficulty in pinpointing compounds with specific roles. The combined effects of natural bioactive substances include synergistic, antagonistic, and independent effects. Antagonistic effect means the inactive compounds contained in these mixes may cause harmful side effects. Thus, the isolation and identification of pure bioactive compounds from *A. cinnamomea* are beneficial to revealing the mechanism underlying the effects of its compounds. Moreover, defining the related mechanisms can improve the use of functional compounds from *A. cinnamomea* in drug development and clinical applications.

## Figures and Tables

**Table 1:** Preparation procedures of polysaccharides from *A. cinnamomea* in recent publications.

Polysaccharides Component	Sources	Extraction Method	Isolation and Purification Method	Reference
ACP	Mycelia	Extracted with cold water	GFC HW65F column (Tosoh Bioscience, 90 cmH × 1.6 cm D); flow rate, 0.4 mL/min	[21]
ACP1	Mycelia	Extracted twice with hot water	DEAE-52 column (300 × 26 mm^2^) and Sephadex G-100 chromatography (1.0 × 80 cm)	[22]
ACP2	Mycelia	Extracted with boiling water for 3 h	DEAE-52 column (300 × 26 mm^2^) and Sephadex G-100 chromatography (1.0 × 80 cm^2^)	[25,32]
ACW0	Mycelia	Extracted with boiling water four times (4 h for each extraction)	DEAE Sepharose Fast Flow and Sephacryl S-100HR systems	[15]
ACPS	Mycelia	Extracted twice in double-distilled water at 95 °C for 4 h	DEAE-52 cellulose anion-exchange column (2.6 cm × 35 cm)	[20]
ACPS-1	Mycelia	Extracted with boiled water for 3 h	DEAE-52 cellulose (2.6 × 20 cm^2^) and Sephadex G-100 column chromatography (1.1 × 100 cm^2^)	[19]
Na10_SPS-F3	Mycelia	0.1 M sodium acetate (pH 5.5) containing 5 mM cysteine, 100 mg papain, and 5 mM EDTA at 60 °C for 24 h	GFC (103 × 1.5 cm^2^ Fractogel BioSeccolumn)	[33]

Note: GFC, gel filtration chromatography; DEAE, dicthylaminoethyl.

**Table 2:** Preparation procedures of triterpenoids from *A. cinnamomea* in recent publications.

Triterpenoids Component	Sources	Extraction Method	Isolation and Purification Method	Reference
Antcin A	Fruiting bodies	Extracted with MeOH at room temperature for 7 days	Silica gel column and semi-preparative HPLC	[48]
Antcin K	Fruiting bodies	Extracted with ethyl acetate for 3 days	Silica gel column and HPLC	[49]
Methylantcinate A	Fruiting bodies	Extracted with n-hexane, chloroform, and methanol under reflux	Silica gel column chromatography and thin-layer chromatography	[50]
Sulphurenic acid	Mycelia	Extracted with methanol at room temperature for 4 days and then partitioned (three times) with ethyl acetate	Silica gel and HPLC	[42]
Dehydroeburicoic Acid	Mycelia	Extracted thrice with methanol at room temperature (4 days × 3)	Silica gel and HPLC	[40]
Eburicoic acid	Mycelia	Extracted thrice with methanol at room temperature (4 days × 3)	Silica gel and HPLC	[41]

**Table 3:** Preparation procedures of ubiquinone derivatives from *A. cinnamomea* in recent publications.

Ubiquinone Derivatives Component	Sources	Extraction Method	Isolation and Purification Method	Reference
Antroquinonol	Mycelia (solid-state)	Extracted three time with n-hexane by stirring at room temperature for 6 h	Silica-gel gravity column (230–400 mesh, 5 × 45 cm^2^) and Sephadex LH-20 (5 × 70 cm^2^)	[56]
4-Acetylantroquinonol B	Mycelia	Extracted with ethyl acetate	HPLC and silica gel column (4.6 × 250 mm^2^)	[62]
Antrocinnamone	Mycelia	Extracted with 95% alcohol	Column chromatography and HPLC	[65]
4-Acetylantrocamol LT3	Mycelia	Extracted with 95% alcohol	Column chromatography and HPLC	[65,66]

**Table 4:** Preparation procedures of maleic and succinic acid derivatives from *A. cinnamomea* in recent publications.

Maleic and Succinic Acid Derivatives	Sources	Extraction Method	Isolation and Purification Method	Reference
Antrodin A	Mycelia	Extracted with absolute ethanol at a ratio of 1:100 (g/mL), the ethanol extract was then extracted twice with ethyl acetate: water = 1:1	Silica gel column chromatography in a Reveleris PREP purification system	[71]
Antrodin C	Mycelia	Extracted in methanol and then partitioned with n-hexane chloroform and ethyl acetate	Silica gel column and semipreparative HPLC	[73]
Antrodin B	Mycelia	extracted with methanol for 24 h at room temperature.	Silica gel column and semipreparative HPLC	[72]

**Table 5:** Preparation procedures of benzene derivatives from *A. cinnamomea* in recent publications.

Benzene Derivatives	Sources	Extraction Method	Isolation and Purification Method	Reference
DMB	Mycelia	Extracted with methanol	Silica gel column and Sephadex LH-20 column	[83]
Antrolone	Mycelia	Extracted with 95% ethanol	silica gel column chromatography and medium pressure liquid chromatography	[79]

**Table 6:** Preparation procedures of glycoprotein from *A. cinnamomea* in recent publications.

Glycoprotein	Sources	Extraction Method	Isolation and Purification Method	Reference
Antrodan	Mycelia	lkali extraction with subsequent acid precipitation.	Sepharose CL-6B column (3.0 × 82 cm^2^) and SEC	[86,87]

## Data Availability

Data is contained within the article.
